# Calcimimetic Prescriptions in Fee-for-Service Medicare Beneficiaries Undergoing Dialysis

**DOI:** 10.1001/jamahealthforum.2025.0452

**Published:** 2025-04-18

**Authors:** Jillian S. Caldwell, Xingxing S. Cheng, Eran Bendavid, Glenn M. Chertow, Darius N. Lakdawalla, Eugene Lin

**Affiliations:** 1 Division of Nephrology, Stanford University School of Medicine, Palo Alto, California; 2 Department of Health Policy, Stanford University School of Medicine, Palo Alto, California; 3 Department of Pharmaceutical and Health Economics, Alfred E. Mann School of Pharmacy and Pharmaceutical Sciences, University of Southern California, Los Angeles; 4 Leonard D. Schaeffer Center for Health Policy and Economics, University of Southern California, Los Angeles; 5 Price School of Public Policy, University of Southern California, Los Angeles; 6 Division of Nephrology, Keck School of Medicine, University of Southern California, Los Angeles

## Abstract

**Importance:**

Calcimimetics are a mainstay of treatment for secondary hyperparathyroidism (sHPT), a ubiquitous condition in end-stage kidney disease (ESKD) associated with fractures, cardiovascular events, and mortality. In 2018, Medicare implemented the Transitional Drug Add-On Payment Adjustment (TDAPA), which shifted calcimimetic coverage from Part D prescription drug plans to Part B. Prior to TDAPA, Medicare beneficiaries with ESKD faced varying out-of-pocket costs for calcimimetics at the point of pharmacy depending on presence and magnitude of low-income subsidies (LISs). TDAPA differentially alleviated barriers to filling these costly medications.

**Objective:**

To assess whether calcimimetic prescriptions increased post-TDAPA among patients subject to high out-of-pocket costs prior to the policy change (patients with Part D coverage without LIS and those lacking Part D coverage).

**Design, Setting, and Participants:**

In this longitudinal cohort study, a difference-in-differences analysis was performed at the patient-quarter level. The sample included adult Medicare fee-for-service beneficiaries undergoing maintenance dialysis between July 1, 2016, and December 31, 2020, at US outpatient dialysis facilities. The US Renal Data System, a national registry of patients with ESKD, was used to collect patient, facility, and claims data. The data analysis occurred between May 2023 and October 2024.

**Exposures:**

LIS extent for patients with Part D coverage (fully subsidized, partially subsidized, not subsidized); presence of Medicare Part D coverage; and whether the patient-quarter was before/after TDAPA implementation.

**Main Outcomes and Measures:**

The main outcome was having 1 or more filled calcimimetic prescriptions per quarter of the study period. A linear regression model was estimated, adjusting for demographics, dialysis modality and access, comorbidities, and facility characteristics, with 2-way fixed effects at the patient and quarter level.

**Results:**

A total of 509 765 adult Medicare fee-for-service beneficiaries were included in the analysis. The cohort had a mean (SD) age of 64 (14) years, was 57% male, 4% Asian, 38% Black, 15% Hispanic, 41% non-Hispanic White, and 3% other race and ethnicity. In adjusted difference-in-differences models, TDAPA’s estimated effect was an absolute increase of 9.8 percentage points (pp) (95% CI, 9.3-10.2 pp) in calcimimetic prescriptions for patients with Part D but no subsidy and a 2.2 pp (95% CI, 1.8-2.6 pp) increase for patients with partial LIS compared to patients with full LIS.

**Conclusions and Relevance:**

The results of this longitudinal cohort study showed that after transitioning calcimimetic coverage from Part D to Part B via TDAPA, calcimimetic prescriptions increased in a graded manner, with the largest increases experienced by patients previously subject to the highest out-of-pocket prescription drug costs. Medicare’s TDAPA policy has the potential to expand access to medications for patients.

## Key Points

**Question:**

Did Medicare’s Transitional Drug Add-On Payment Adjustment (TDAPA) increase calcimimetic prescriptions for patients with end-stage kidney disease undergoing maintenance dialysis?

**Findings:**

In a difference-in-differences analysis of more than 500 000 Medicare beneficiaries, compared to patients with Medicare Part D coverage and full cost-sharing subsidies, the TDAPA policy was followed by a 2–percentage point increase in filled quarterly calcimimetic prescriptions in patients with partial cost-sharing subsidies and a 10–percentage point increase in patients with no subsidies.

**Meaning:**

Medicare policy has the potential to expand access to medications for patients with end-stage kidney disease.

## Teaser

This cohort study assesses whether calcimimetic prescriptions increased following the Transitional Drug Add-On Payment Adjustment policy among patients subject to high out-of-pocket costs.

## Introduction

Nearly all of the 550 000 patients with end-stage kidney disease (ESKD) undergoing maintenance dialysis have secondary hyperparathyroidism (sHPT), a condition associated with fractures, cardiovascular events, and mortality.^1,2,3^ Managing sHPT involves decreasing parathyroid hormone secretion with activated vitamin D, calcimimetics (cinacalcet or etelcalcetide), and/or surgical parathyroidectomy.^4^ Approximately 30% to 35% of patients receive calcimimetics for the management of sHPT.^5^ Before calcimimetics, many patients required parathyroidectomies because they could not safely receive vitamin D due to hypercalcemia. Calcimimetics do not cause hypercalcemia and allow patients to avoid parathyroidectomies; the parathyroidectomy rate in ESKD dropped from approximately 6.0 to 7.5 per 1000 patients prior to the introduction of calcimimetics to approximately 3.5 to 5.0 per 1000.^6,7,8^

In fee-for-service (FFS) Medicare (traditional Medicare or Medicare Parts A and B), most dialysis-related medications are bundled into a Part B, per-dialysis treatment payment. Medications without an intravenous equivalent are excluded from the bundle and instead covered FFS by Part D stand-alone prescription drug plans.^9^ Before 2018, the only commercially available calcimimetic was oral cinacalcet, which was thus covered under Part D. With a sticker price estimated at $600 to $700 monthly, many patients with prescription drug coverage were responsible for sizeable copays when obtaining calcimimetics.^3,10,11^ Although the low-income subsidy (LIS) offers copay assistance, many with ESKD do not qualify.^5^ When faced with high out-of-pocket costs and limited budgets, patients forego medications, and patients without LIS are especially likely to experience cost-related nonadherence.^12,13,14,15^

Etelcalcetide, an intravenous calcimimetic, was approved by the US Food and Drug Administration in 2017, prompting the Centers for Medicare & Medicaid Services (CMS) to transition calcimimetic payment (oral and intravenous) from Part D to Part B per-treatment payment bundle. The resulting transition period from January 2018 to December 2020 was known as the Transitional Drug Add-on Payment Adjustment (TDAPA).^16^ Under TDAPA, calcimimetics were paid FFS outside the dialysis bundle by Part B, which is mandatory, instead of Part D. In 2021, TDAPA ended for calcimimetics, and they were incorporated into Part B dialysis bundled payments.^17^

TDAPA removed cost-sharing barriers at the point of pharmacy, transitioned the dispensing of calcimimetics from pharmacies to dialysis units, and coincided with the introduction of a new therapy for management of sHPT. Because patients with varying degrees of LIS experienced differential cost-sharing prior to TDAPA, the policy provided an opportunity to examine the extent to which out-of-pocket costs at the point of pharmacy dissuaded patients from obtaining costly medications. We hypothesized that TDAPA increased calcimimetic prescriptions proportional to the degree of pre-TDAPA cost-sharing, accounting for temporal and patient-level factors.

## Methods

This cohort study was approved by the Stanford Institutional Review Board, which granted waivers of informed consent because this was a secondary data analysis deemed to have minimal risk to participants. We adhered to Strengthening the Reporting of Observational Studies in Epidemiology (STROBE) reporting guidelines.

We used data from the United States Renal Data System (USRDS), a national registry of US patients with ESKD, which contains (1) Medicare Part A and Part B claims for patients enrolled in FFS Medicare^5^; (2) the Consolidated Renal Operations in a Web-Enabled Network (CROWNWeb), a patient-month dataset of facility-reported clinical measures, including vascular access type; and (3) the annual dialysis facility survey, which includes dialysis facility characteristics. We supplemented USRDS data with CMS’ Dialysis Facility Compare annual files.^18^

### Study Population

We constructed a longitudinal, patient-quarter–level panel dataset. We identified adult (≥18 years old) Medicare FFS beneficiaries undergoing dialysis in the US at any time between July 1, 2016, and December 31, 2020, to allow for 1.5 years of pre-TDAPA data (the policy began on January 1, 2018) and 3 years of postpolicy data. The TDAPA payment ended on December 31, 2020, corresponding to the end date of the most up-to-date files from the USRDS at the time of analysis. Approximately 55% to 65% of patients undergoing dialysis between 2016 and 2020 were FFS Medicare beneficiaries; the remainder had employer-sponsored insurance (ESI) or Medicare Advantage (MA).^5^ During the study period, the FFS population with ESKD was younger, with more Black patients, fewer Hispanic patients, and more dual Medicare-Medicaid eligible patients compared to the ESI/MA population.^5,19^ To maximize comorbidity capture, patients contributed quarters only when they had at least 1 year of consecutive FFS Medicare coverage. We excluded patient-quarters with missing Part D enrollment information or missing dialysis facility data and censored patients for death or transplant (eFigure 1 in Supplement 1). We included patients with kidney transplants who returned to dialysis following graft failure. See the eMethods in Supplement 1 for the technical appendix.

### Exposures

We conducted a difference-in-differences analysis. Conceptually, our primary analysis examined cost-sharing barriers to prescription drugs by comparing the change in calcimimetic prescriptions pre-TDAPA/post-TDAPA among patients with different degrees of LIS. LIS was categorized as full subsidy (100% premium subsidy and no copayment), partial subsidy (100% or partial premium subsidy and partial copayment), no subsidy (Part D but no LIS), or not enrolled in Part D based on the majority LIS category in each quarter. We treated LIS full subsidies (ie, patients with no cost-sharing) as control patients. Prior to TDAPA, patients’ out-of-pocket costs for calcimimetics were proportionate to subsidy level: With full subsidies, annual median (IQR) was $0 ($0.00-0.00); with partial subsidies, $10.80 ($6.60-19.80); and without subsidies, $657.88 ($141.00-1791.19). The USRDS does not contain out-of-pocket costs from Part B claims, so post-TDAPA calcimimetic costs could not be ascertained.

In a secondary analysis to estimate the effect of expanding prescription drug coverage or shifting existing drug coverage through non-Medicare sources to Medicare, we conducted pre-TDAPA/post-TDAPA comparisons among patients who did not have Part D coverage compared to the control group, composed of patients with Part D coverage. A limitation of this analysis is that we could not observe instances where patients received calcimimetics pre-TDAPA through sources other than Part D (eg, free medication samples or employer-sponsored prescription drug plans).

Because Black patients tend to have more severe sHPT than persons of other racial and ethnic backgrounds, we tested for heterogeneous treatment effects in a triple differences analysis (ie, subgroup analysis by race and ethnicity).^20,21,22^ Race and ethnicity in the USRDS are determined from the Medical Evidence 2728 form, which may be filled out (ie, designated) by nephrologists, social workers, or other dialysis unit staff with or without patients’ input. The race and ethnicity categories included Asian, Black, Hispanic, non-Hispanic White, and other, which consisted of American Indian or Alaska Native, Native Hawaiian or Pacific Islander, other or multiracial, and unknown.

### Main Outcomes

The primary outcome was presence of a calcimimetic prescription during a patient-quarter. From July 1, 2016, to December 31, 2017, calcimimetic prescriptions were identified using Medicare Part D claims. From January 1, 2018, to December 31, 2020, calcimimetic prescriptions were identified using Part B dialysis claims via Healthcare Common Procedure Coding System codes J0604 and J0606.

### Covariates

We controlled for patient-level demographics (age and dual eligibility for Medicare and Medicaid during that quarter), dialysis modality (in-center hemodialysis, home hemodialysis, or peritoneal dialysis), dialysis access type (arteriovenous fistula, arteriovenous graft, central venous catheter, or peritoneal catheter), duration of ESKD, and dialysis facility characteristics (for-profit status, freestanding vs hospital-based facility, and ESRD network).^23^ We did not adjust for dual Medicare-Medicaid eligibility because it was collinear with LIS. We included comorbidities (updated annually) using the *International Classification of Diseases, Ninth Revision (ICD-9)* and *International Statistical Classification of Diseases and Related Health Problems, Tenth Revision (ICD-10)* codes from inpatient and outpatient claims as described by Elixhauser et al.^24^ Data were missing in less than 2% of all covariates. We incorporated patient-level fixed effects (a unique intercept for each patient), which control for fixed time-invariant characteristics, including sex and primary reason for ESKD. We also included time fixed effects (indicators for quarter) that control for shared temporal trends in the outcomes among groups.

### Statistical Analysis

We conducted a difference-in-differences analysis to determine whether calcimimetic prescriptions in patients with partial or no LIS changed pre-TDAPA/post-TDAPA. Conceptually, the model compared the difference in calcimimetic prescriptions pre-TDAPA/post-TDAPA among patients with less than full Part D subsidies (the first difference) to the same difference among patients with full subsidies (the second difference). The resulting difference-in-differences can be interpreted as the estimated change in calcimimetic prescriptions resulting from the TDAPA policy after accounting for unobserved, temporal utilization patterns affecting the entire population. Results are reported as absolute percentage-point (pp) differences.

Formally, we used linear regression with 2-way fixed effects. The dependent variable was calcimimetic prescriptions in each patient-quarter, and the independent variable of interest was the interaction between LIS level (or Part D coverage) and whether the quarter was post-TDAPA.^25^ We incorporated patient-level and quarter-level fixed effects.

To assess for heterogeneous effects of TDAPA on calcimimetic prescriptions by race and ethnicity, we conducted a triple differences analysis by incorporating a full set of interactions among race and ethnicity, LIS level (or Part D coverage), and whether the quarter was post-TDAPA (omitting interactions absorbed into the fixed effects). We estimated the marginal effect of TDAPA on calcimimetic prescriptions for each racial and ethnic group.

The difference-in-differences model only estimates the average change in calcimimetic prescriptions pre-TDAPA/post-TDAPA. To analyze longitudinal trends in the policy effect, we conducted an event study, where we interacted LIS level (or Part D coverage) with each quarter-level fixed effect. We assessed for parallel trends by examining the significance of the interaction terms for pre-TDAPA quarters. All models used cluster-robust standard errors at the facility level.

We reported baseline characteristics by LIS level and Part D coverage in the pre-TDAPA and post-TDAPA periods as the percentage of individuals in each group. To assess for covariate balance, we conducted individual unadjusted difference-in-differences regressions with each covariate as the outcome. The resulting estimates compare the extent each covariate changed pre-TDAPA/post-TDAPA when stratifying patients by LIS level (or Part D coverage). We considered 2-sided *P* values less than .05 as statistically significant. Data were analyzed from May 2023 to October 2024 using SAS statistical software, version 14.3 (SAS Institute), and STATA, version 17.0 (StataCorp LLC).

## Results

### Baseline Characteristics

The cohort comprised 509 765 unique FFS Medicare beneficiaries and 4 435 058 patient-quarters between July 1, 2016, and December 31, 2020 (eFigure 1 in Supplement 1). The mean (SD) patient age was 64 (14) years; 57% were male, 4% Asian, 38% Black, 15% Hispanic, 41% non-Hispanic White, and 3% other race and ethnicity, as defined in the Methods. Patients without LIS were more likely to be male, non-Hispanic White, younger than 65 years, undergoing peritoneal dialysis, and have a shorter duration of ESKD; these patients were also less likely to be dual Medicare-Medicaid eligible, have diabetes, heart failure, and peripheral vascular disease compared to patients with partial or full subsidies (Table).

**Table.** Cohort Characteristics Before and After Transitional Drug Add-On Payment Adjustment (TDAPA) Policy Implementation by Low-Income Subsidy Level^a^

Characteristic	Patient-quarters, No. (%)							
	Pre-TDAPA (n = 1 459 603)				Post-TDAPA (n = 2 975 445)			
	Non–Part D	No subsidy	Partial subsidy	Full subsidy	Non–Part D	No subsidy	Partial subsidy	Full subsidy
Total	227 525 (16)	367 255 (25)	646 109 (44)	218 714 (15)	460 437 (15)	765 543 (26)	1 284 948 (43)	464 527 (16)
Sex								
Female	77 875 (34)	145 635 (40)	294 185 (46)	118 418 (54)	155 930 (34)	299 306 (39)	576 926 (45)	247 676 (53)
Male	149 650 (66)	221 620 (60)	351 924 (54)	100 296 (46)	304 507 (66)	466 237 (61)	708 022 (55)	216 851 (47)
Age group, y								
<45	10 366 (5)	9484 (3)	117 347 (18)	14 152 (6)	20 392 (4)	18 471 (2)	318 542 (17)	30 082 (6)
45-64	78 108 (34)	86 643 (24)	335 355 (52)	79 815 (37)	156 011 (34)	171 895 (22)	661 148 (51)	167 902 (36)
>65	139 051 (61)	271 128 (74)	193 407 (30)	124 747 (57)	284 034 (62)	575 177 (75)	405 258 (32)	266 543 (57)
Race and ethnicity								
Asian	9180 (4)	10 878 (3)	26 955 (4)	8084 (4)	19 014 (4)	25 514 (3)	54 842 (4)	18 865 (4)
Black	76 301 (34)	102 020 (28)	287 168 (44)	93 751 (43)	153 413 (33)	195 400 (26)	545 222 (42)	194 548 (42)
Hispanic	19 831 (9)	27 083 (7)	135 379 (21)	33 046 (15)	39 881 (9)	59 445 (8)	269 750 (21)	67 993 (15)
Non-Hispanic White	114 238 (50)	222 212 (61)	176 741 (27)	77 599 (35)	230 019 (50)	473 406 (62)	372 398 (29)	169 749 (37)
Other^b^	7975 (4)	5062 (1)	20 136 (3)	6234 (3)	18 110 (4)	11 778 (2)	43 736 (3)	13 375 (3)
Dual Medicare-Medicaid eligibility	NA	3240 (1)	513 501 (79)	185 195 (85)	NA	8454 (1)	1 010 940 (79)	393 538 (85)
Origin of ESKD								
Diabetic kidney disease	96 557 (42)	157 922 (43)	276 329 (43)	121 365 (56)	197 316 (43)	333 159 (44)	561 706 (44)	257 268 (55)
Hypertensive kidney disease	67 284 (30)	112 967 (31)	203 869 (32)	57 794 (26)	139 246 (30)	236 398 (31)	406 880 (32)	125 764 (27)
Glomerulonephritis	2783 (12)	38 089 (10)	80 315 (12)	13 746 (6)	54 075 (12)	76 244 (10)	151 075 (12)	28 474 (6)
Polycystic kidney disease	8166 (4)	11 813 (3)	16 322 (3)	2970 (1)	16 188 (4)	25 603 (3)	34 494 (3)	6698 (1)
Other, unknown cause	26 912 (12)	45 691 (12)	68 198 (11)	22 390 (10)	51 874 (11)	91 891 (12)	128 393 (10)	45 398 (10)
Duration of ESKD, y								
<3	56 761 (25)	108 527 (30)	145 264 (22)	52 362 (24)	106 362 (23)	220 486 (29)	268 975 (21)	102 379 (22)
3-5	84 788 (37)	131 562 (36)	216 367 (33)	72 733 (33)	174 238 (38)	281 478 (37)	431 787 (34)	156 991 (34)
>5	85 976 (38)	127 169 (35)	284 478 (44)	93 619 (43)	179 837 (39)	263 579 (34)	584 186 (45)	205 156 (44)
Dialysis modality								
In-center hemodialysis	196 613 (86)	321 936 (88)	589 933 (91)	211 892 (97)	394 466 (86)	659 806 (86)	1 166 362 (91)	448 528 (97)
Home hemodialysis	6982 (3)	7545 (2)	9359 (1)	1508 (1)	15 445 (3)	17 687 (2)	22 360 (2)	4308 (1)
Peritoneal dialysis	23 930 (11)	37 774 (10)	46 817 (7)	5314 (2)	50 526 (11)	88 050 (12)	96 226 (7)	11 691 (3)
Dialysis access								
Arteriovenous fistula	142 308 (63)	233 563 (64)	416 168 (64)	125 780 (58)	279 667 (61)	476 053 (62)	819 437 (64)	265 452 (57)
Arteriovenous graft	37 272 (16)	61 905 (17)	124 613 (19)	51 229 (24)	73 472 (16)	122 841 (16)	239 612 (19)	102 767 (22)
Catheter	19 546 (9)	30 814 (8)	53 670 (8)	33 919 (16)	43 615 (10)	71 438 (9)	120 710 (9)	79 842 (17)
Peritoneal catheter	23 930 (11)	37 774 (10)	46 817 (7)	5314 (2)	50 526 (11)	88 050 (12)	96 226 (8)	11 691 (3)
Comorbidities								
Congestive heart failure	97 563 (43)	180 182 (49)	286 585 (44)	134 176 (61)	208 954 (45)	396 595 (52)	615 294 (48)	298 740 (64)
Peripheral vascular disease	61 767 (27)	117 576 (32)	164 771 (26)	97 353 (45)	113 441 (25)	223 437 (29)	301 491 (23)	188 111 (41)
Diabetes	132 751 (58)	233 887 (64)	396 513 (61)	173 523 (79)	273 295 (59)	498 860 (65)	812 448 (63)	373 611 (80)
Cancer	26 741 (12)	49 619 (14)	44 692 (7)	18 873 (9)	54 950 (12)	109 418 (14)	93 098 (7)	41 757 (9)
Coronary artery disease	109 663 (48)	205 298 (56)	281 864 (44)	134 437 (62)	225 900 (49)	435 387 (57)	585 107 (46)	291 903 (63)
Type of dialysis facility								
For-profit	202 849 (89)	329 319 (90)	579 538 (90)	194 936 (89)	408 243 (89)	678 147 (89)	1 142 060 (89)	408 764 (88)
Freestanding	218 584 (96)	355 849 (97)	628 300 (97)	210 678 (96)	438 360 (95)	730 914 (95)	1 236 653 (96)	440 123 (95)

Abbreviations: ESKD, end-stage kidney disease; NA, not applicable.

^a^ Data were missing in less than 2% of all covariates; percentages may not sum to 100% due to rounding.

^b^ American Indian, Alaska Native, Native Hawaiian, Pacific Islander, other, multiracial, or unknown.

Unadjusted difference-in-differences models of each covariate to assess covariate balance showed significant associations between TDAPA and age, duration of ESKD, dialysis modality, dialysis access type, heart failure, peripheral vascular disease, diabetes, cancer, coronary artery disease, and dialyzing at a for-profit dialysis facility (eTable 1 in Supplement 1). However, these effect estimates were small on a pp basis. Baseline characteristics and unadjusted difference-in-difference estimates by Part D coverage showed similar patterns (eTable 2 in Supplement 1).

### Estimated Effect of TDAPA on Calcimimetic Prescriptions (Difference-in-Differences) by LIS

**Figure 1.**
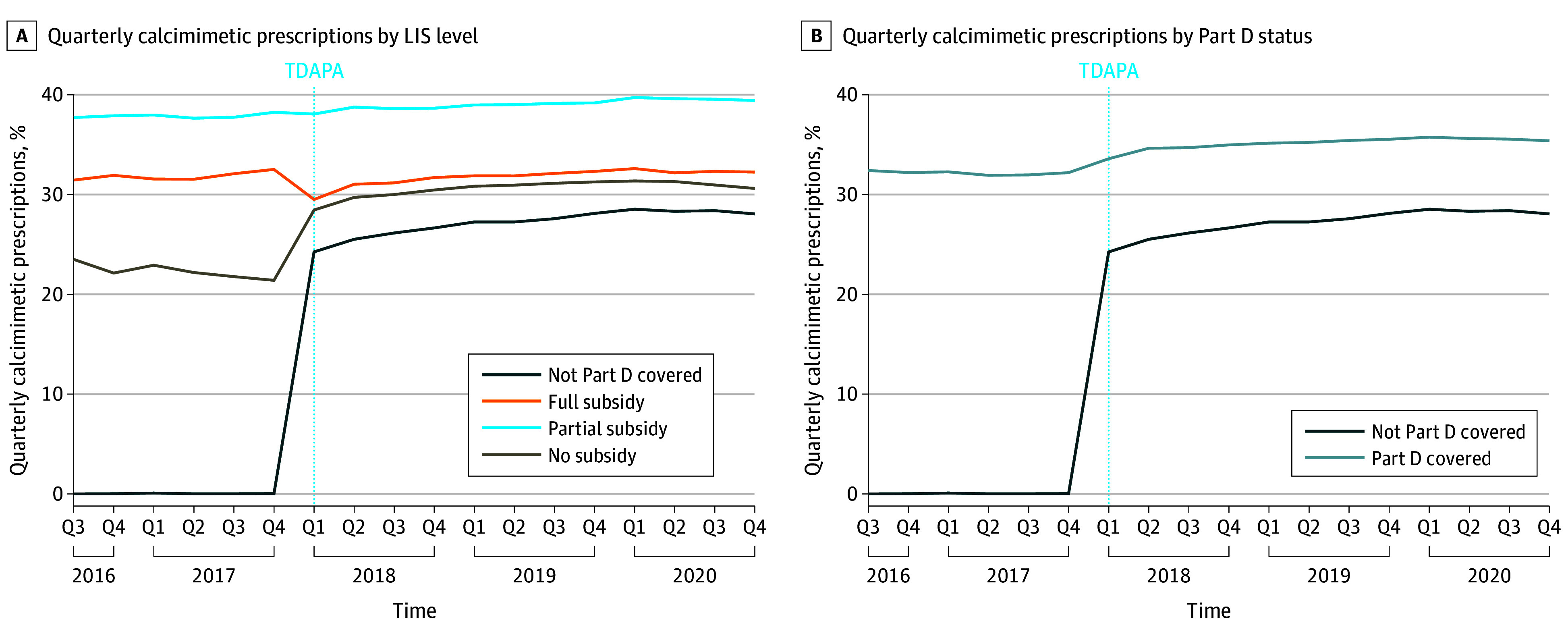
Unadjusted Calcimimetic Prescriptions Before and After Transitional Drug Add-On Payment Adjustment (TDAPA) by Low-Income Subsidy (LIS) Level and Part D Coverage Status Unadjusted quarterly calcimimetic prescriptions before and after TDAPA. The dashed blue vertical line indicates the time of TDAPA policy implementation. The proportion of patients receiving at least 1 calcimimetic prescription in a quarter using Part D claims (for quarters before January 1, 2018) and monthly Part B dialysis claims (for quarters in and after January 1, 2018), stratified by LIS level (A) and Part D coverage status (B). Q indicates quarter.

Immediately following the passage of TDAPA (ie, in quarter 1 of 2018), quarterly calcimimetic prescriptions changed in a pattern inversely related to LIS amount: unadjusted, a 7.1-pp increase for patients with Part D but no LIS, a 0.2-pp decrease in patients with a partial LIS, and a 3.0-pp decrease in patients with full LIS (Figure 1A). In patients with Part D coverage but no LIS and patients without Part D coverage, the increase was primarily due to increased cinacalcet prescriptions rather than etelcalcetide prescriptions (eFigure 2A-2B in Supplement 1).

**Figure 2.**
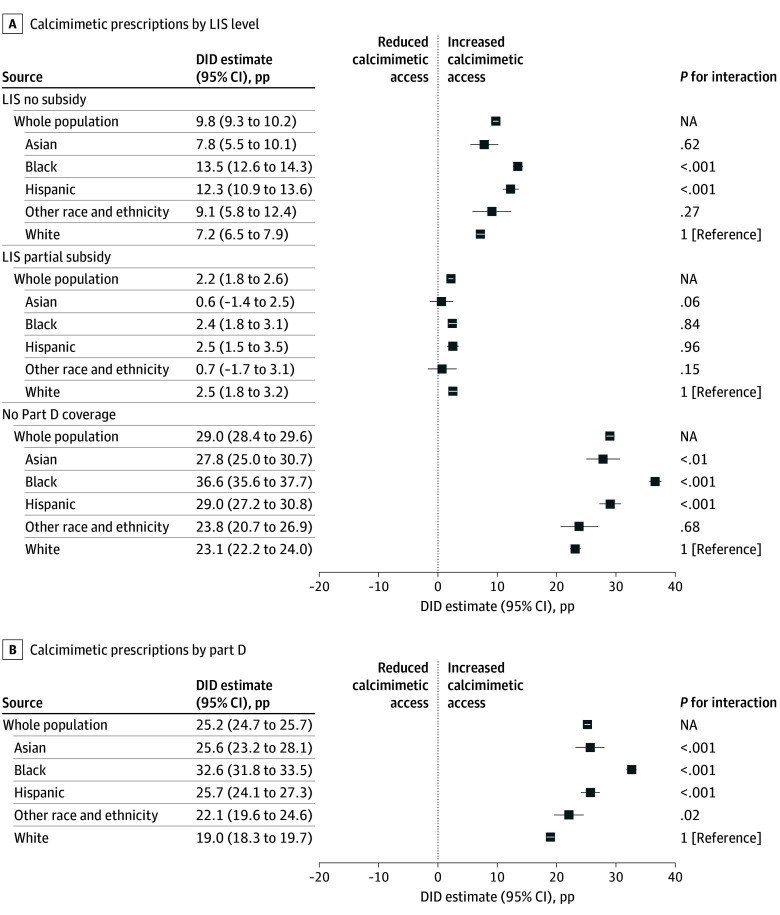
Estimated Effect of Transitional Drug Add-On Payment Adjustment (TDAPA) on Calcimimetic Prescriptions by Low-Income Subsidy (LIS) Level, Part D Coverage Status, and Race and Ethnicity The estimated effect of TDAPA on calcimimetic prescriptions by LIS level (A) and Part D status (B). Estimates for the whole population represent difference-in-differences (DID) estimates. Estimates for each designated race and ethnicity category represent difference-in-difference-in-difference estimates. DID estimates represent absolute percentage-point (pp) differences in calcimimetic prescriptions compared to the reference group. Estimates greater than 0 represent increased calcimimetic prescriptions following TDAPA compared to before TDAPA. Race and ethnicity were designated by clinicians with or without patient input. The other race and ethnicity category includes American Indian or Alaska Native, Native Hawaiian or Pacific Islander, other or multiracial, and unknown. NA indicates not applicable.

**Figure 3.**
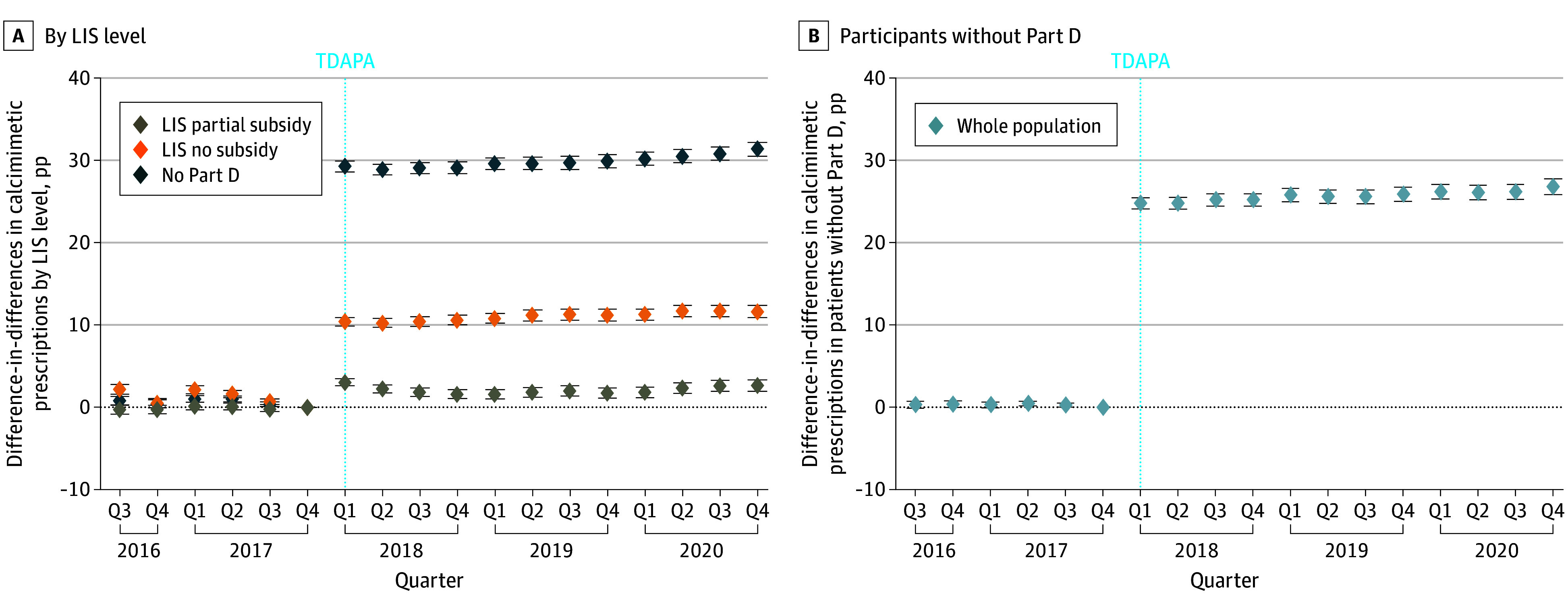
Event Study Estimates of Calcimimetic Prescriptions Before and After Transitional Drug Add-On Payment Adjustment (TDAPA) by Low-Income Subsidy (LIS) Level and Part D Coverage Status Event study estimates of the effect of TDAPA on calcimimetic prescriptions by LIS level (A) and Part D coverage status (B), obtained by interacting LIS subsidy level (or Part D coverage) with each quarter-level fixed effect of the study period. Estimates represent absolute percentage-point (pp) differences in calcimimetic prescriptions compared to the reference group (either full LIS [A] or Part D–covered individuals [B]) in each quarter of the study period, and error bars represent 95% CIs. Estimates greater than 0 represent increased calcimimetic prescriptions following TDAPA compared to before TDAPA. The dashed blue vertical line indicates the time of TDAPA policy implementation. Q indicates quarter.

After adjusting for confounders, the estimated effect of TDAPA was a graded increase in calcimimetic prescriptions: As LIS increased, the rise in prescriptions attenuated. When compared to patients with the full LIS, TDAPA was followed by a 9.8-pp increase (95% CI, 9.3-10.2 pp) in patients with Part D but no LIS, a 2.2-pp increase (95% CI, 1.8-2.6 pp) in patients with a partial LIS, and a 29.0-pp increase (95% CI, 28.4-29.6 pp) in calcimimetic prescriptions in patients with no Part D coverage (Figure 2A). The event study similarly demonstrated a significant and persistent increase in calcimimetic prescriptions immediately after TDAPA implementation, with the same pattern (Figure 3A). Although pre-TDAPA period regression coefficients were statistically significant, they were near zero (≤2 pp for all pre-TDAPA quarters), suggesting roughly parallel trends in calcimimetic prescriptions between groups.

### Estimated Effect of TDAPA on Calcimimetic Prescriptions (Difference-in-Differences) by Part D Coverage

Between quarter 4 of 2017 and quarter 1 of 2018, calcimimetic prescriptions increased by 1.4 pp among patients with Part D coverage and by 24.2 pp among those without Part D (Figure 1B). In the adjusted difference-in-differences model, TDAPA was followed by a 25.2-pp increase (95% CI, 24.7-25.7 pp) in calcimimetic prescriptions in patients without Part D coverage (Figure 2B). The event study demonstrated a similar pp increase after TDAPA implementation, which was sustained throughout the TDAPA period (Figure 3B).

### Differences by Race and Ethnicity (Triple Differences)

**Figure 4.**
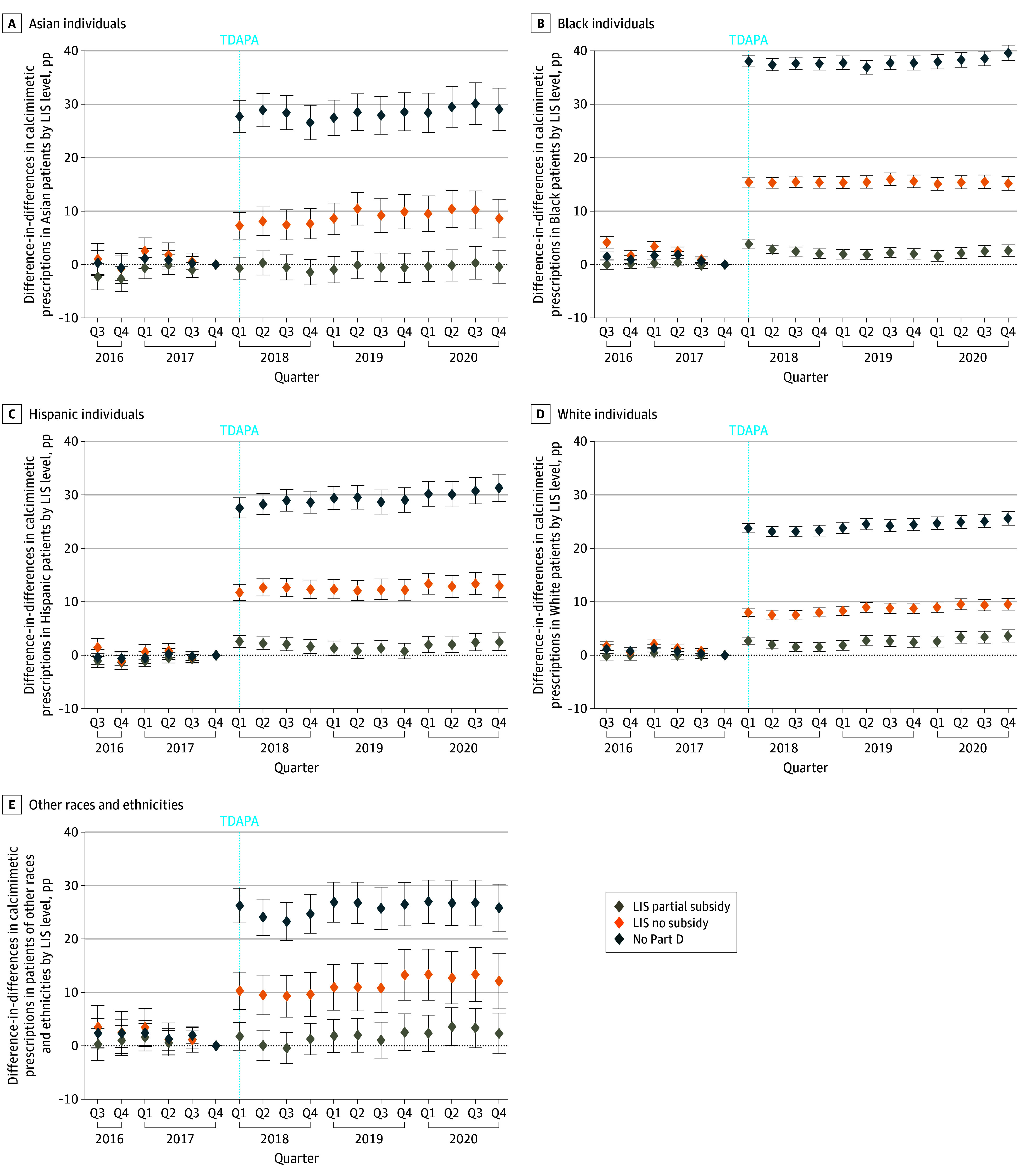
Event Study Estimates of Calcimimetic Prescriptions Before and After Transitional Drug Add-On Payment Adjustment (TDAPA) by Low-Income Subsidy (LIS) Level and Race and Ethnicity Event study estimates of calcimimetic prescriptions before and after TDAPA by LIS level and beneficiary race and ethnicity, obtained by interacting LIS level with each quarter-level fixed effect of the study period and race and ethnicity. Estimates for patients designated as Asian (A), Black (B), Hispanic (C), non-Hispanic White (D), and other race and ethnicity (E) by clinicians with or without patient input. Estimates represent absolute percentage-point (pp) differences in calcimimetic prescriptions compared to the reference group (non-Hispanic White patients) in each quarter of the study period, and error bars represent 95% CIs. Estimates greater than 0 represent increased calcimimetic prescriptions following TDAPA compared to before TDAPA. The dashed blue vertical line indicates the time of the TDAPA policy implementation. The other race and ethnicity category includes American Indian or Alaska Native, Native Hawaiian or Pacific Islander, other or multiracial, and unknown. Q indicates quarter.

Significant differences in findings by race and ethnicity were observed when comparing patients with Part D coverage without subsidies and patients without Part D coverage to patients with full subsidies, but not when comparing partial to full subsidies. Following TDAPA, Black patients had the highest increase in calcimimetic prescriptions (patients without subsidies: 13.5 pp [95% CI, 12.6-14.3 pp]; *P* for interaction < .001; patients without Part D coverage: 36.6 pp [95% CI, 35.6-37.7 pp]; *P* for interaction < .001), followed by Hispanic patients (patients without subsidies: 12.3 pp [95% CI, 10.9-13.6 pp]; *P* for interaction < .001; patients without Part D coverage: 29.0 pp [95% CI, 27.2-30.8 pp]; *P* for interaction < .001) when compared with non-Hispanic White patients (patients without subsidies [reference group]: 7.2 pp [95% CI, 6.5-7.9 pp]; patients without Part D coverage [reference group]: 23.1 pp [95% CI, 22.2-24.0 pp]; Figure 2A, Figure 4A-E).

There were significant differences across race and ethnicity groups when comparing patients with and without Part D coverage in a similar pattern. TDAPA was followed by the largest increase in calcimimetic prescriptions among Black patients, followed by Hispanic and Asian patients, and patients of other racial and ethnic backgrounds when compared with non-Hispanic White patients (Figure 2B; eFigure 3 in Supplement 1).

## Discussion

In this longitudinal cohort study, we estimated that the TDAPA add-on payment increased calcimimetic prescriptions by 2 pp to 10 pp among FFS Medicare beneficiaries undergoing maintenance dialysis with Part D coverage, depending on the magnitude of LIS subsidies. The increase in calcimimetic prescriptions was highest among Black and Hispanic patients (as designated by clinicians with or without patient input).

There are several potential explanations for the increase in calcimimetic prescriptions. The TDAPA policy differentially alleviated patients’ exposure to cost-sharing at the point of pharmacy. We note a graded increase in calcimimetic prescriptions following TDAPA, with patients subject to the highest out-of-pocket costs prior to the policy experiencing the largest increase in calcimimetic prescriptions. This is consistent with prior work demonstrating that patients forego medications (either in part or fully) when faced with large prescription drug costs and that patients with LIS are less likely to experience cost-related nonadherence.^12,14,15^ TDAPA could also have reduced patients’ out-of-pocket expenses; however, owing to data limitations, we cannot report out-of-pocket costs for calcimimetic prescriptions from the post-TDAPA period. Notably, patients were likely not informed of their out-of-pocket costs for calcimimetics at the time of their dialysis treatments. This contrasts with the pharmacy setting, where patients were directly presented with cost-sharing obligations.

Post-TDAPA expenses are likely heterogeneous. Part B involves 20% coinsurance for covered drugs, but this is waived for patients with dual Medicare-Medicaid coverage (most with partial and full subsidies in our cohort were dually eligible; Table) and may be reduced for patients with Medigap plans.^26,27^ Manufacturer rebates could have lowered calcimimetic costs for dialysis facilities. Finally, dialysis facilities often forgive patients’ unpaid out-of-pocket costs because Medicare reimburses facilities up to 65% of this bad debt.^28^ Thus, post-TDAPA out-of-pocket costs and the extent to which patients paid them remain unclear. Nevertheless, some patients may have been harmed if cost-sharing increased from $0 under Part D to a 20% copayment under Part B, potentially contributing to the slight decrease in calcimimetic prescriptions in patients with full LIS following TDAPA (Figure 1A).

Following TDAPA, dialysis units directly dispensed calcimimetics, alleviating difficulties associated with self-medication management. Pill burden, medication regimen complexity, and lack of transportation to pharmacies contribute to high rates of nonadherence in patients undergoing maintenance dialysis.^29,30^ Additionally, Black and Hispanic patients disproportionately live in neighborhoods with fewer pharmacies (“pharmacy deserts”) compared with White neighborhoods, which may explain why Black and Hispanic patients experienced the greatest increases in calcimimetic prescriptions following TDAPA in our study.^31^

Calcimimetic prescriptions could have increased because etelcalcetide was made available by the policy or because the introduction of etelcalcetide generated momentum in an area of clinical inertia, prompting clinicians to simultaneously prescribe more cinacalcet and etelcalcetide. Unadjusted calcimimetic prescriptions illustrate that patients with Part D coverage without LIS and patients without Part D coverage primarily experienced an increase in cinacalcet prescriptions (eFigure 2A-2B in Supplement 1). Additionally, because etelcalcetide was introduced equally to exposed (patients without LIS or with partial LIS) and unexposed (patients with full LIS) groups, our difference-in-difference estimates are unlikely driven by the new therapy. Finally, add-on payments may have incentivized clinicians to prescribe these medications when they might not have prescribed them otherwise.

The largest increase in calcimimetic prescriptions was in Black patients across LIS levels. We cannot explain this differential increase, in part because our data lack markers of sHPT severity such as serum parathyroid hormone concentrations. In general, sHPT is more severe in Black compared with White patients, a disparity that may reflect biological differences in calcium and parathyroid hormone signaling, inadequate control of sHPT, longer duration of undiagnosed kidney disease, younger age at ESKD onset, and reduced access to transplantation.^20,21,32^ Alternatively, Black and Hispanic patients could have benefited most from TDAPA through removal of cost-sharing barriers or moving calcimimetic administration from pharmacies to dialysis centers, as noted previously.

We estimated that newly expanding coverage or shifting existing coverage through non-Medicare sources to Medicare via TDAPA increased calcimimetic prescriptions among patients without Part D by nearly 25 pp. This represents an upper bound, as pre-TDAPA estimates of calcimimetic prescriptions among patients without Part D coverage are, by default, zero because data limitations preclude observation of alternate sources of prescription drug fills, such as free samples or ESI. Of note, the Medicare Payment Advisory Committee estimates that fewer than 10% of Medicare beneficiaries have prior employer coverage.^33^

Our findings have broad implications for access to medications in the ESKD population. The stated purpose of TDAPA is to provide a transition period to collect data on the use of new dialysis medications, to assess potential benefits in practice, and to inform updates to the bundled dialysis price such that new therapies are appropriately compensated post-TDAPA.^34^ Understanding TDAPA’s effectiveness is critical because CMS transitioned phosphate binders and other phosphate-lowering therapies (oral medications used to correct hyperphosphatemia, another disturbance of bone and mineral metabolism) from Part D to Part B via TDAPA as of January 1, 2025.^35^ These data suggest that TDAPA may improve access to therapies for patients with ESKD.

Following the end of the TDAPA period, coverage for calcimimetics transitioned to inclusion in dialysis bundled payments, which could have reduced incentives to use these drugs compared to add-on payments. Additionally, the end of TDAPA coincided with the 21st Century Cures Act, which lifted the prohibition on patients with ESKD enrolling in MA and resulted in a large influx of MA enrollment. Anecdotally, some health care organizations providing dialysis note difficulty funding these medications for patients on MA plans.^36^ The USRDS does not include claims from MA beneficiaries undergoing dialysis, so we could not observe calcimimetic use in MA.

### Limitations

Limitations of our study include the observational nature of our data. Our population is limited to FFS beneficiaries and excludes patients with ESI and MA. The difference-in-differences analysis and 2-way fixed-effects model allow us to control for some unobserved confounders (eg, time-invariant patient characteristics and contemporaneous trends correlated with calcimimetic use), but residual confounding may be present. Our formal tests of parallel trends were statistically significant; however, the effect size was near zero and unlikely to be clinically meaningful. As mentioned previously, owing to data limitations, we could not observe markers of sHPT severity. We did not evaluate changes in other treatments for sHPT, such as the use of vitamin D analogues or parathyroidectomies.

## Conclusions

This large national cohort study demonstrated that the TDAPA policy resulted in increased access to calcimimetics for patients undergoing maintenance dialysis. The magnitude of increase was larger for patients who faced large out-of-pocket costs when filling medications prior to the policy and for Black and Hispanic patients. These findings suggest that insurance coverage expansion could increase access to more costly medications in the ESKD population.

## Notes

Supplement 1. eMethods eFigure 1. Cohort Sampling Scheme eTable 1. Regression Coefficients on Covariates by LIS Level eTable 2. Cohort Characteristics by Part D Status eFigure 2. Unadjusted Cinacalcet and Etelcalcetide Prescriptions Before and After Implementation of TDAPA, by LIS Level and Part D Status eFigure 3. Event Study Estimates of Calcimimetic Prescriptions Before and After TDAPA by Part D Coverage Status and Designated Race/Ethnicity

Supplement 2. Data Sharing Statement
