# Supplementary material for: Calcimimetic Prescriptions in Fee-for-Service Medicare Beneficiaries Undergoing Dialysis
Source: JAMA Health Forum. 2025 Apr 18;6(4):e250452. doi: 10.1001/jamahealthforum.2025.0452 (PMC12008760; doi:10.1001/jamahealthforum.2025.0452)
Supplement: Supplement 1. — eMethods eFigure 1. Cohort Sampling Scheme eTable 1. Regression Coefficients on Covariates by LIS Level eTable 2. Cohort Characteristics by Part D Status eFigure 2. Unadjusted Cinacalcet and Etelcalcetide Prescriptions Before and After Implementation of TDAPA, by LIS Level and Part D Status eFigure 3. Event Study Estimates of Calcimimetic Prescriptions Before and After TDAPA by Part D Coverage Status and Designated Race/Ethnicity [file jamahealthforum-e250452-s001.pdf]

Supplemental Online Content

Caldwell JS, Cheng XS, Bendavid E, Chertow GM, Lakdawalla DN, Lin E. Calcimimetic prescriptions in fee-for-service Medicare beneficiaries undergoing dialysis. *JAMA Health Forum.* 2025;6(4):e250452. doi:10.1001/jamahealthforum.2025.0452

eMethods

eFigure 1. Cohort Sampling Scheme

eTable 1. Regression Coefficients on Covariates by LIS Level

eTable 2. Cohort Characteristics by Part D Status

eFigure 2. Unadjusted Cinacalcet and Etelcalcetide Prescriptions Before and After Implementation of TDAPA, by LIS Level and Part D Status

eFigure 3. Event Study Estimates of Calcimimetic Prescriptions Before and After TDAPA by Part D Coverage Status and Designated Race/Ethnicity

This supplemental material has been provided by the authors to give readers additional information about their work.

eMethods.

1.1 Difference-in-Differences Estimator

Our difference-in-differences model used a panel dataset at the patient-quarter level, indexed for patient i in quarter q. We used a two-way fixed effects model, which included unique patient-level and quarter-level intercepts.[1] The former adjusted for unobserved patient-level confounders invariant to time. The latter adjusted for unobserved trends at the national level. Conceptually, the model examined the change in calcimimetic use before and after TDAPA among patients who did not have Part D coverage. Formally, we conducted the following linear regression:

$$Y_{iq} = \alpha_q + \alpha_i + \beta_{NoPartD} * I_{NoPartD,iq} + \gamma_{DiD} * I_{NoPartD,iq} * PostTDAPA_q + B_X * X_{iq} + \varepsilon_{iq}$$

where:

i indexes each person
q indexes each quarter
Y_{iq} is the outcome of interest (i.e., whether patient i used calcimimetics in quarter q)
α_q is a vector of quarter fixed effects (i.e., a unique intercept for each quarter q)
α_i is a vector of patient fixed effects (i.e., a unique intercept for each person i)
$I_{NoPartD,iq}$ is a binary variable indicating whether person i did not have Part D coverage in quarter q
$PostTDAPA_q$ is a binary variable indicating whether quarter q occurred on or after 1/1/2018[2]
X_{iq} is a vector of time-varying control variables for person I, including facility-level and regional-level characteristics[3]
$\beta_{NoPartD}$, γ_{DiD}, B_X are coefficients. γ_{DiD} is the coefficient of interest (the difference-in-differences estimator)
ε_{iq} is a random, white noise error term

---

[1] We use fixed effects instead of random effects because fixed effects are allowed to be correlated with the outcome <u>and</u> the other independent variables. We do not make any functional form assumptions for fixed effects.
[2] $PostTDAPA_q$ is excluded from the model because it is colinear with the quarter fixed effects
[3] Characteristics that are time invariant (e.g., sex) are absorbed into the person fixed effects and excluded from X_{iq}. Race/ethnicity, which is time invariant, was excluded from the difference-in-differences estimator. In the triple differences estimator, race/ethnicity interacted with time variables are not time invariant, and thus these interactions were included in the model.

© 2025 Caldwell JS et al. *JAMA Health Forum*.

The regression was estimated using cluster-robust standard errors at the person-level. When conducting analyses comparing the association between TDAPA and calcimimetic use for patients with different levels of LIS, the $I_{NoPartD,iq}$ variable was replaced with a categorical variable designating the level of LIS subsidy for person i in quarter q.

1.2 Triple Differences Estimator

The triple differences estimator interacted race/ethnicity with the presence of Part D coverage and whether the quarter was before or after TDAPA. Specifically, we estimated the following model:

$$Y_{iq} = \alpha_q + \alpha_i + \beta_{NoPartD} * I_{NoPartD,iq} + \beta_{post,race} * PostTDAPA_q * race_i + \beta_{NoPartD,race} * I_{NoPartD,iq} * race_i + \gamma_{DiD,White} * I_{NoPartD,iq} * PostTDAPA_q + \gamma_{DiD,race} * I_{NoPartD,iq} * PostTDAPA_q * race_i + B_X * X_{iq} + \varepsilon_{iq}$$

where the variables are defined identical to previous. Additionally:

$race_i$ is the self-declared race or ethnicity of person i

$\gamma_{DiD,White}$ is the association between TDAPA and calcimimetic use for White patients, which is the reference category for this analysis.

$\gamma_{DiD,race}$ is the triple difference, or the additional policy association between TDAPA and calcimimetic use for non-White patients.

To obtain the association between TDAPA and calcimimetic use for non-White patients, we obtain the linear combination of $\gamma_{DiD,White}$ and $\gamma_{DiD,race}$. The triple differences analysis was also conducted for different levels of LIS subsidy, which replaced the indicator for Part D coverage.

1.3 Event Study

© 2025 Caldwell JS et al. *JAMA Health Forum*.

To conduct the event study, we interacted the indicator for Part D coverage with the individual quarter fixed effects. By convention, we omitted the interaction for the quarter immediately prior to TDAPA (the quarter corresponding to 10/1/2017 to 12/31/2017, which is indexed by t = –1 below). Specifically, we conducted the following linear regression:

$$Y_{iq} = \alpha_q + \alpha_i + \beta_{NoPartD} * I_{NoPartD,iq} + \sum_{t=-6}^{-2} \left[\gamma_{pre,t} * 1(q=t) * I_{NoPartD,iq}\right]$$

$$+ \sum_{t=0}^{T} \left[\gamma_{post,t} * 1(q=t) * I_{NoPartD,iq}\right] + B_X * X_{iq} + \varepsilon_{iq}$$

where:

T is the last quarter of the study (the quarter 10/1/2020 to 12/31/2020)

t runs from quarter -6 (the beginning of the study, or the quarter corresponding to 7/1/2016 to 9/30/2016) to T. The start of TDAPA (the quarter from 1/1/2018 to 3/31/2018) is indexed to t = 0

$1(q = t)$ is an indicator function that equals 1 when $q = t$ and 0 when $q \neq t$

$\gamma_{pre,t}$ are the pre-policy coefficients of interest and are used to assess the parallel trends assumption

$\gamma_{post,t}$ are the post-policy coefficients of interest and represent the "time-varying" difference-in-differences estimator

We conducted generalized versions of this model to conduct event studies by LIS subsidy and by racial/ethnic subgroup.

1.4 Classifying Low-Income Subsidy (LIS)

LIS was categorized as full subsidy (100% premium subsidy and no copayment), partial subsidy (100% or partial premium subsidy and partial copayment), no subsidy (Part D but no LIS), or not enrolled in Part D based on the USRDS Part D enrollment files (CST_SHR_GRP_CD

© 2025 Caldwell JS et al. *JAMA Health Forum*.

variables). Patients with dual Medicare-Medicaid eligibility and missing subsidy information were classified as receiving full LIS. Patients with Part D coverage and missing subsidy information were classified as receiving no LIS. Patients without Part D coverage were classified as receiving no LIS. We categorized LIS level on the majority of months in the quarter; ties went to the greater subsidy level.

1.5 Identifying Calcimimetic Prescriptions

Part D Claims (July 1, 2016-December 31, 2017)

Calcimimetic prescriptions in the Part D Claims was assessed using the Part D files. When processing claims for the years 2016, 2019, and 2020, generic names for cinacalcet and etelcalcetide were used to identify calcimimetic use. In 2017 and 2018, only NDC codes (no generic names) were available in the part D claims. A key was generated using 2016 and 2019 claims to identify all NDC codes associated with cinacalcet or etelcalcetide and used to find claims corresponding to calcimimetics.

At least one prescription fill in each quarter was counted as calcimimetic use for that quarter. Based on the number of days' supply associated with the prescription fill, we generated end dates for each prescription. If the end date for a prescription extended into the following quarter, both quarters were marked as having calcimimetic use. We stopped counting calcimimetic fills via Part D claims on December 31, 2017 to avoid double-counting after implementation of TDAPA.

Part B Claims (January 1, 2018-December 31, 2020)

Calcimimetic use in the Part B claims was assessed using revenue and physician supplier files for dialysis facilities (type of bill 72) in outpatient, dialysis, or skilled nursing facility claims. HCPCS codes J0604 (cinacalcet) and J0606 (etelcalcetide) were used to identify calcimimetic

© 2025 Caldwell JS et al. *JAMA Health Forum*.

use. At least one claim for either of these HCPCS codes during the quarter was counted as calcimimetic present.

1.6 Identifying Comorbidities Using the Elixhauser Algorithm

We ascertained all comorbid conditions using ICD-9 and ICD-10 diagnosis and procedure codes from the Elixhauser Comorbidity software algorithm (Agency for Healthcare Research and Quality, v2020.1) in any position requiring at least 1 inpatient or 2 outpatient encounters separated by at least 1 day.[1,2] Additional comorbidities of coronary artery disease, cerebral vascular disease, smoking, dyslipidemia, left ventricular hypertrophy, and arrhythmias were assessed using this method using the corresponding ICD-9 and ICD-10 codes.

We assessed baseline comorbidities in 2016 with a look-back window to January 1, 2014. We updated comorbidities annually with the same look-back window extending to January 1, 2014. We excluded the comorbidities of renal failure, fluid and electrolyte disorders, and deficiency anemias since these are universal in patients on maintenance dialysis from our regression. Additional covariates not described in Elixhauser were ascertained to enhance comorbidity completeness: coronary artery disease, cerebral vascular disease, smoking, dyslipidemia, left ventricular hypertrophy, and arrhythmia.

© 2025 Caldwell JS et al. *JAMA Health Forum*.

eFigure 1. Cohort Sampling Scheme

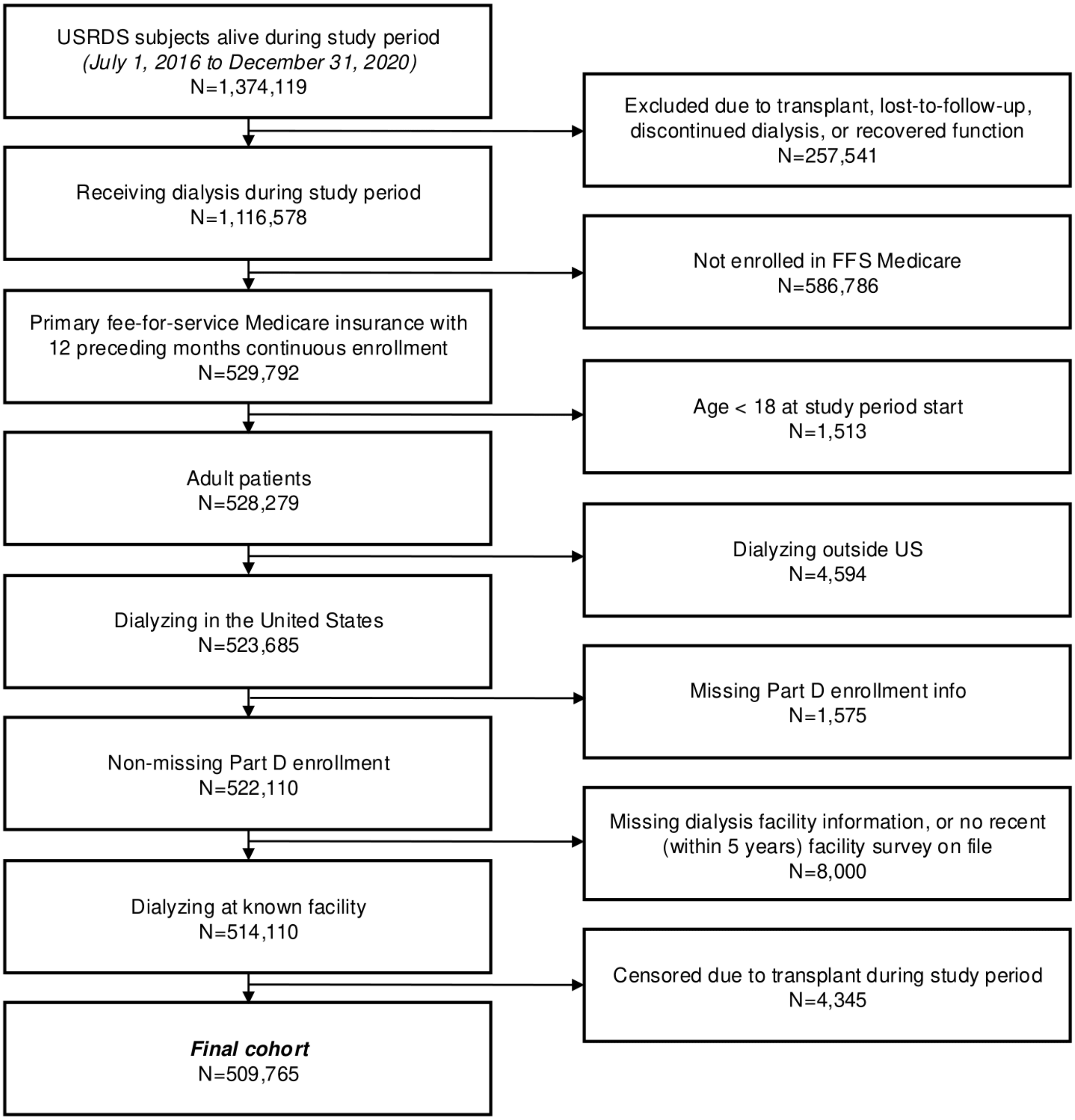

© 2025 Caldwell JS et al. *JAMA Health Forum*.

eTable 1. Baseline Characteristics, by LIS Status

	Pre-TDAPA				Post-TDAPA		
	**Not Part D Covered** N=55,152	**No Subsidy** N=87,758	**Partial Subsidy** N=144,400	**Full Subsidy** N=53,954	**No Subsidy** N=123,082	**Partial Subsidy** N=182,763	**Full Subsidy** N=77,653
**Sex (% Male)**	66%	60%	54%	46%	61%	55%	47%
**Age**							
<45 years old	5%	3%	18%	6%	2%	17%	6%
45-64 years old	34%	34%	52%	37%	22%	51%	36%
>65 years old	61%	74%	30%	57%	75%	32%	57%
**Race / Ethnicity (%)**							
-Non-Hispanic White	50%	61%	27%	35%	62%	29%	37%
-Black	34%	28%	44%	43%	26%	42%	42%
-Asian	4%	3%	4%	4%	3%	4%	4%
-Hispanic	9%	7%	21%	15%	8%	21%	15%
-American Indian, Alaska Native, Native Hawaiian, Pacific Islander, mixed race	4%	1%	3%	3%	2%	3%	3%
**Etiology of ESKD (%)**							
-Diabetic kidney disease	42%	43%	43%	56%	44%	44%	55%
-Hypertensive kidney disease	30%	31%	32%	26%	31%	32%	27%
-Glomerulonephritis	12%	10%	12%	6%	10%	12%	6%
-Polycystic kidney disease	4%	3%	3%	1%	3%	3%	1%
-Other, unknown cause	12%	12%	11%	10%	12%	10%	10%
**Duration of ESKD (%)**							
<3 years	25%	30%	22%	24%	29%	21%	22%
3-5 years	37%	36%	33%	33%	37%	34%	34%
>5 years	37%	35%	44%	43%	34%	45%	44%
**Dialysis Modality (%)**							
-In-center Hemodialysis	86%	88%	91%	97%	86%	91%	97%
-Home hemodialysis	3%	2%	1%	1%	2%	2%	1%
-Peritoneal dialysis	11%	10%	7%	2%	12%	7%	3%
**Dialysis Access (%)**							
-Arteriovenous fistula	63%	64%	65%	58%	63%	64%	58%
-Arteriovenous graft	16%	17%	19%	24%	16%	19%	22%
-Catheter	9%	8%	8%	16%	9%	9%	17%
-Peritoneal catheter	11%	10%	7%	2%	12%	8%	3%
**CHF (%)**	44%	49%	45%	62%	52%	48%	64%
**Peripheral Vascular Disease (%)**	28%	32%	26%	45%	29%	24%	41%
**Diabetes (%)**	60%	64%	62%	80%	65%	63%	81%
**Malignancy (%)**	12%	14%	7%	9%	14%	7%	9%
**Coronary Artery Disease (%)**	50%	56%	44%	62%	57%	46%	63%
**For-profit dialysis facility (%)**	89%	90%	90%	89%	89%	89%	88%
**Freestanding dialysis facility (%)**	96%	97%	97%	96%	95%	96%	95%

eTable 1. Baseline characteristics of the cohort by LIS category. No Part D coverage group shown for reference; for change from baseline in the no part D group, refer to Table 1 in the

© 2025 Caldwell JS et al. *JAMA Health Forum*.

main manuscript. Percentages may not sum to 100% due to rounding. ESKD, end-stage kidney disease; LIS, low-income subsidy; TDAPA, transition drug add-on payment adjustment.

© 2025 Caldwell JS et al. *JAMA Health Forum*.

eTable 2. Regression Coefficients on Covariates by LIS

	**No Subsidy**		**Partial Subsidy**		**Full Subsidy**	
	**DiD Estimate**	**p-value**	**DiD Estimate**	**p-value**	**DiD Estimate**	**p-value**
**Sex (% Male)**	-	-	-	-	-	-
**Age**						
<45 years old	0.005	0.000	-0.022	0.000	-0.001	0.185
45-64 years old	-0.004	0.003	0.020	0.000	-0.008	0.000
>65 years old	-0.001	0.396	0.001	0.271	0.010	0.000
**Race / Ethnicity (%)**	-	-	-	-	-	-
**Etiology of ESKD (%)**	-	-	-	-	-	-
**Duration of ESKD (%)**						
<3 years	-0.045	0.000	0.020	0.000	-0.002	0.529
3-5 years	0.027	0.000	0.006	0.088	0.063	0.000
>5 years	-0.019	0.000	-0.026	0.000	-0.025	0.000
**Dialysis Modality (%)**						
-In-center Hemodialysis	0.003	0.005	-0.006	0.000	-0.11	0.000
-Home hemodialysis	-0.001	0.027	0.000	0.785	-0.002	0.001
-Peritoneal dialysis	-0.002	0.071	0.006	0.000	0.013	0.000
**Dialysis Access (%)**						
-Arteriovenous fistula	-0.001	0.649	-0.007	0.000	-0.020	0.000
-Arteriovenous graft	0.002	0.139	0.003	0.008	-0.007	0.000
-Catheter	0.003	0.801	-0.003	0.019	0.015	0.000
-Peritoneal catheter	-0.002	0.114	0.006	0.000	0.013	0.000
**CHF (%)**	0.005	0.065	-0.144	0.000	-0.031	0.000
**Peripheral Vascular Disease (%)**	-0.000	0.904	-0.012	0.000	-0.029	0.000
**Diabetes (%)**	-0.006	0.001	-0.003	0.099	-0.011	0.000
**Malignancy (%)**	-0.003	0.077	-0.004	0.007	-0.004	0.043
**Coronary Artery Disease (%)**	-0.003	0.267	-0.006	0.010	-0.024	0.000
**For-profit dialysis facility (%)**	-0.000	0.966	-0.002	0.001	-0.001	0.320
**Freestanding dialysis facility (%)**	-0.000	0.622	-0.000	0.791	0.001	0.258

eTable 2. Regression coefficients on covariates by LIS. DiD estimates represent the unadjusted differences-in-differences regression with each covariate as the outcome to assess for covariate balance. The resulting estimate compares the extent each covariate changed pre-/post-TDAPA when stratifying patients by Part D coverage. Time-invariant patient-level characteristics (sex, race/ethnicity, and etiology of ESKD) are absorbed into person-level fixed effects and therefore not reported. DiD, differences-in-differences; ESKD, end-stage kidney disease; TDAPA, transitional drug add-on payment adjustment.

© 2025 Caldwell JS et al. *JAMA Health Forum*.

eFigure 2. Unadjusted Cinacalcet and Etelcalcetide Prescriptions by Part D Coverage

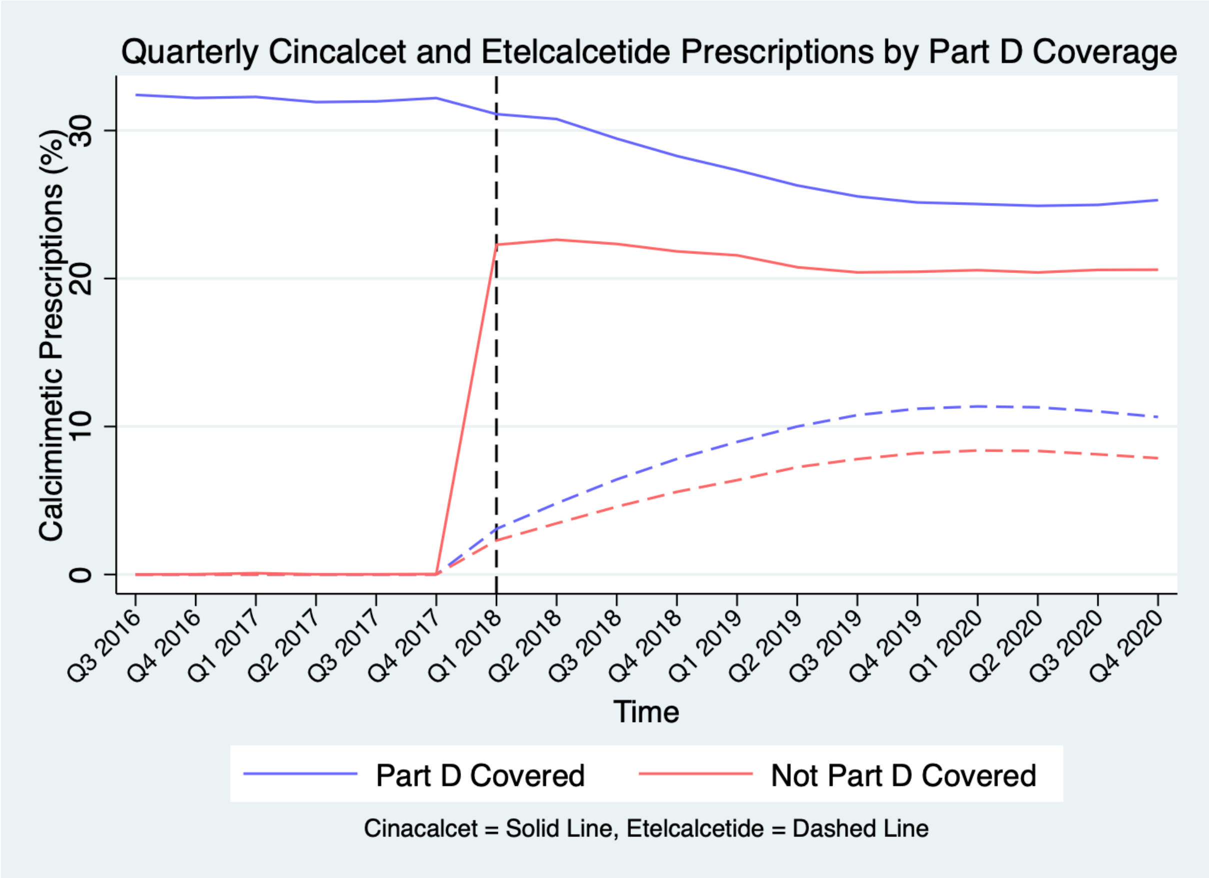

eFigure 2. Unadjusted calcimimetic prescriptions before and after TDAPA. Proportion of patients receiving at least one cinacalcet (solid line) or etelcalcetide (dashed line) prescription in a quarter using Part D claims (for quarters prior to January 1, 2018) and monthly Part B dialysis claims (for quarters in and after January 1, 2018), stratified by Part D coverage status (A) and low-income subsidy status (B). TDAPA, transitional drug add-on payment adjustment.

© 2025 Caldwell JS et al. *JAMA Health Forum*.

eFigure 3. Event Study Estimates of Calcimimetic Prescriptions Before and After TDAPA by LIS and Race/Ethnicity

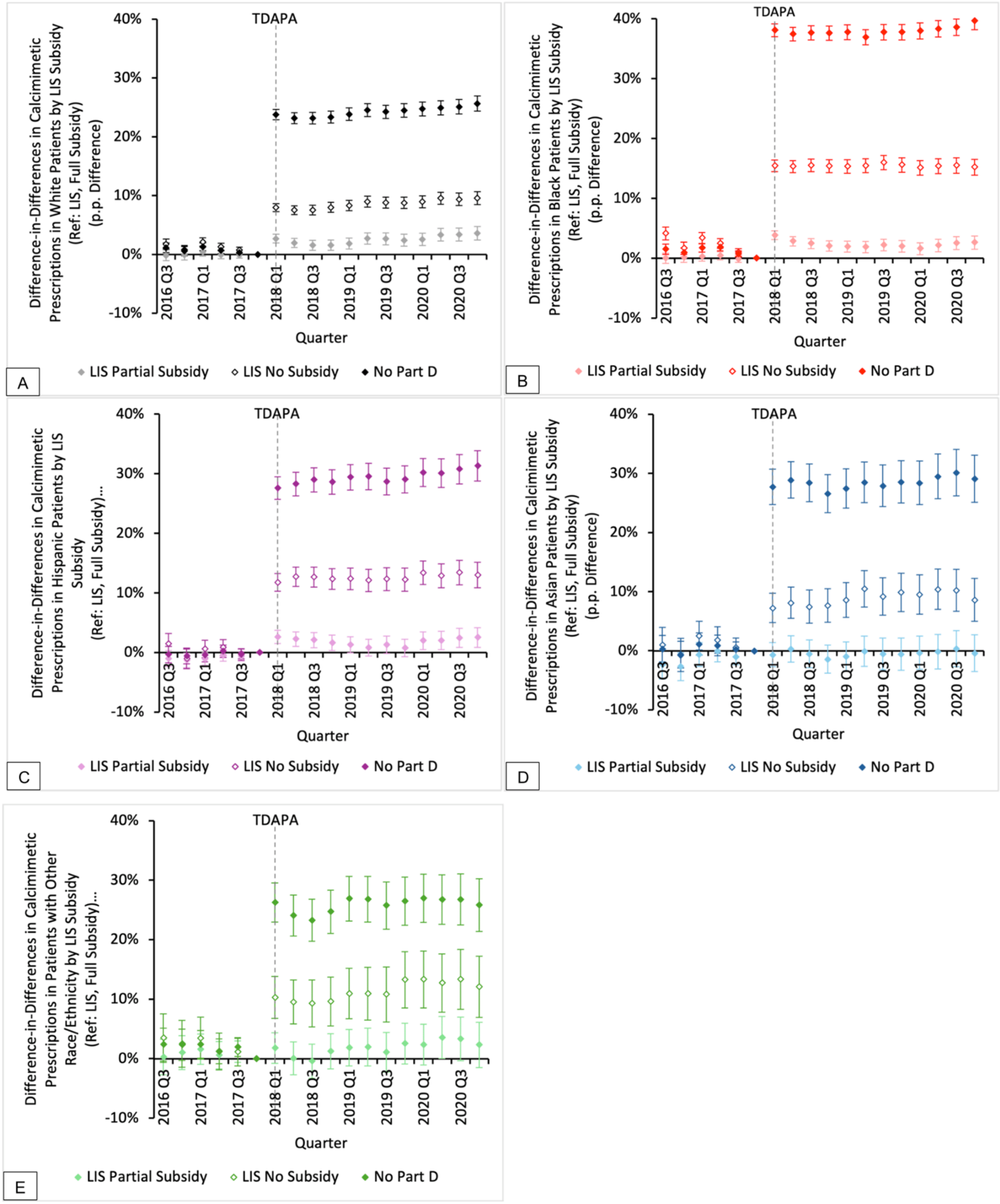

© 2025 Caldwell JS et al. *JAMA Health Forum*.

eFigure 3. Event study estimates of calcimimetic prescriptions before and after TDAPA by LIS and race/ethnicity, obtained by interacting LIS subsidy level with each quarter-level fixed effect of the study period. Estimates for patients designated as non-Hispanic. White (A) Black (B), Hispanic (C), Asian (D) and other race/ethnicity (E). LIS, low-income subsidy; TDAPA, transitional drug add-on payment adjustment.

© 2025 Caldwell JS et al. *JAMA Health Forum*.
