# Supplementary material for: Calcimimetic Prescriptions in Fee-for-Service Medicare Beneficiaries Undergoing Dialysis
Source: JAMA Health Forum. 2025 Apr 18;6(4):e250452. doi: 10.1001/jamahealthforum.2025.0452 (PMC12008760; doi:10.1001/jamahealthforum.2025.0452)
Supplement: Supplement 2. — Data Sharing Statement [file jamahealthforum-e250452-s002.pdf]

Data Sharing Statement

Caldwell. Calcimimetic Prescriptions in Fee-for-Service Medicare Beneficiaries Undergoing Dialysis. *JAMA Health Forum*. Published April 18, 2025.
doi:10.1001/jamahealthforum.2025.0452

Data
Data available: No

Additional Information
Explanation for why data not available: Requires DUA.
